# Supplementary material for: Munc13-1 and Munc18-1 together prevent NSF-dependent de-priming of synaptic vesicles
Source: Nat Commun. 2017 Jun 21;8:15915. doi: 10.1038/ncomms15915 (PMC5482055; doi:10.1038/ncomms15915)
Supplement: Supplementary Information [file ncomms15915-s1.pdf]

Type of file: PDF
Size of file: 0 KB
Title of file for HTML: Supplementary Information
Description: Supplementary Figures and Supplementary Table.

Type of file: PDF
Size of file: 0 KB
Title of file for HTML: Peer Review File
Description:

Supplementary Figure. 1

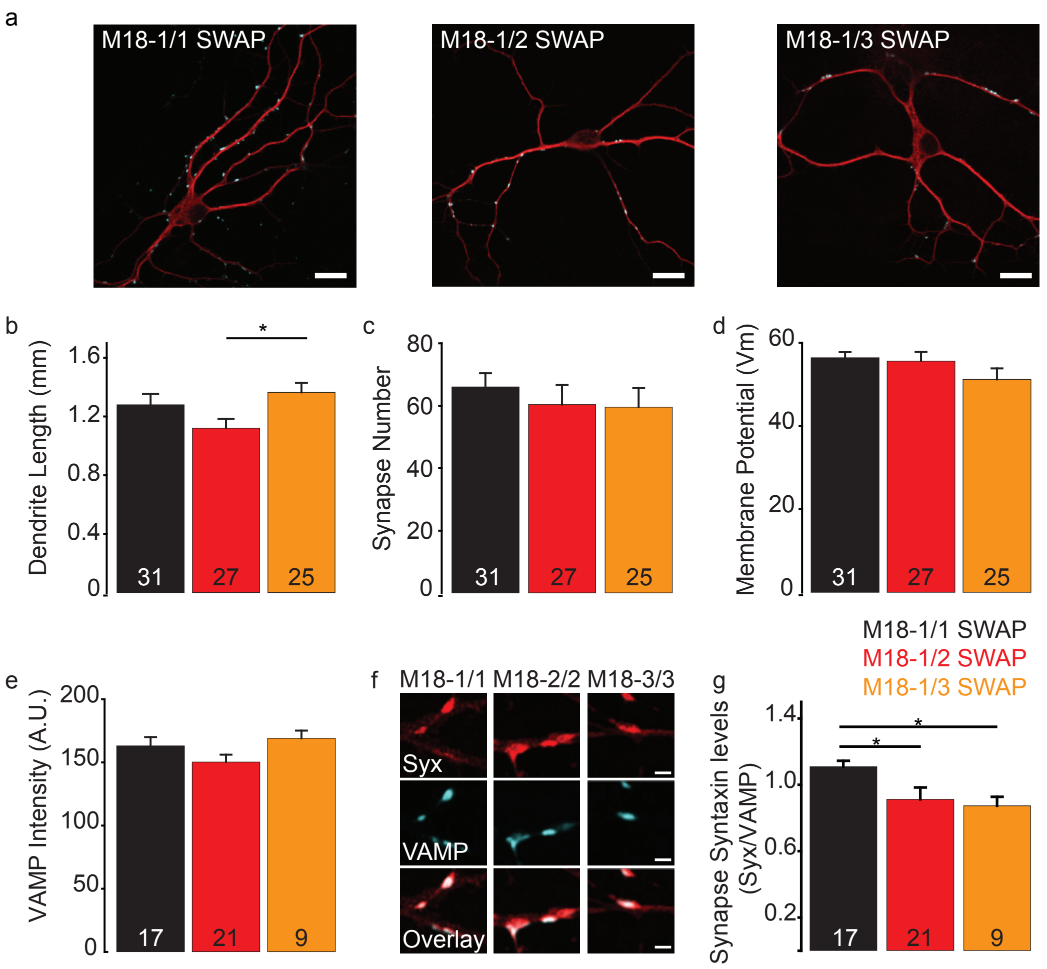

Supplementary Figure1
Viral expression of Munc18-2 or -3 rescues munc18-1 null mutant neurons and restores neuronal viability and morphology

(a) Typical examples of null mutant neurons infected with M18-1, -2, -3 viral particles at DIV1 and stained for MAP2 (dendritic maker) and synapsin (presynaptic terminal marker) at DIV14. Scale bar represents 50 µm.
(b, c) Total dendrite length (b, synapse number (c) and membrane potential (d) of autaptic null mutant neurons expressing M18-1, -2, -3.
(e, f, g) VAMP/synaptobrevin expression level (e), example of syntaxin and VAMP co-staining (f) and syntaxin levels compared to VAMP in autaptic null mutant neurons expressing M18-1, -2, -3. Scale bar represents 2 µm.
All data in this figure are means ± SEMs; $*p < 0.05$, $**p < 0.01$, $***p < 0.001$ as determined by ANOVA. See Table S1 for all values, SEMs and n-numbers plotted in this figure.

Supplementary Figure. 2

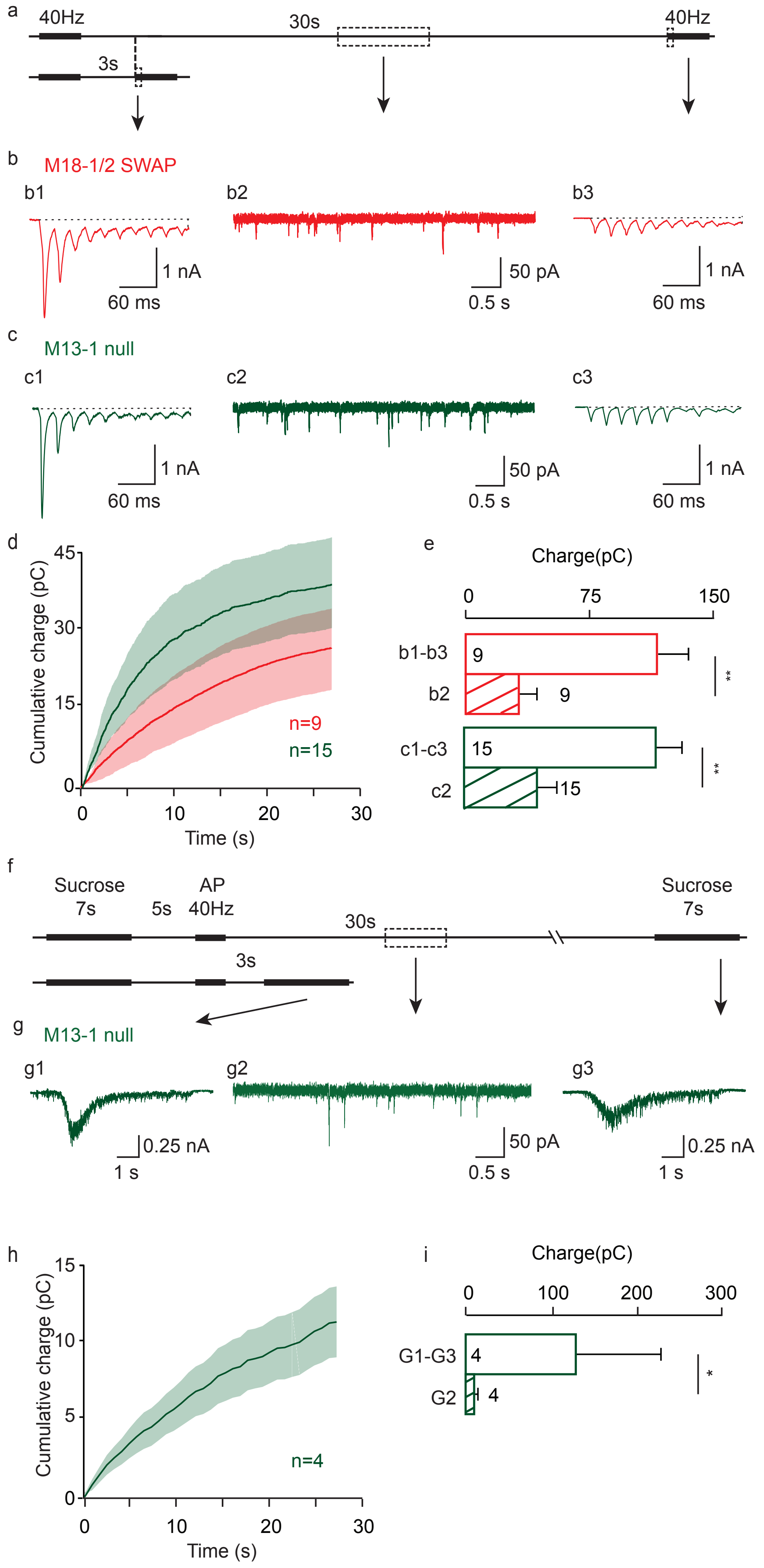

Supplementary Figure2

Primed vesicles rapidly de-prime in the absence of Munc13-1

(a) Paradigm to assess de-priming by dual 40Hz train stimulation (see methods for details).

(b, c) sample traces of dual 100 stimuli at 40Hz with 3s or 30s interval in *munc18-1/2SWAP* (b) and *munc13-1 null* (c) synapses.

(d) Spontaneous fusion events were quantified for the 3-30s intervals.

(e) To quantify de-priming, the total charge of the first 10 responses (b1, b3; c1, c3) of each dual 100 stimuli at 40Hz was quantified. The spontaneous fusion of vesicles in the 3-30s intervals (b2; c2) cannot explain the loss of fusion-competent vesicles after 30s, defined as the difference in total charge between 3s and 30s intervals (b1-b3; c1-c3).

(f) Paradigm to assess de-priming by dual 500 mM hypertonic sucrose application.

(g) Sample traces of dual sucrose application with 3s or 30s interval in *munc13-1 null* synapses. (h) Spontaneous fusion events were quantified for the 3-30s intervals.

(h) Spontaneous fusion events were quantified for the 3 - 30s intervals.

(i) To quantify de-priming, the total charge of the sucrose responses (g1, g3) after 100 stimuli at 40Hz was quantified. The spontaneous vesicle fusion in the 3-30s intervals (g2) cannot explain the loss of fusion-competent vesicles after 30s, defined as the difference in total charge between 3s and 30s intervals (g1-g3).

All data in this figure are means ± SEMs; p value determined by Wilcoxon signed rank test. See supplementary Table 1 for all values, SEMs and n-numbers plotted in this figure.

Supplementary Figure. 3

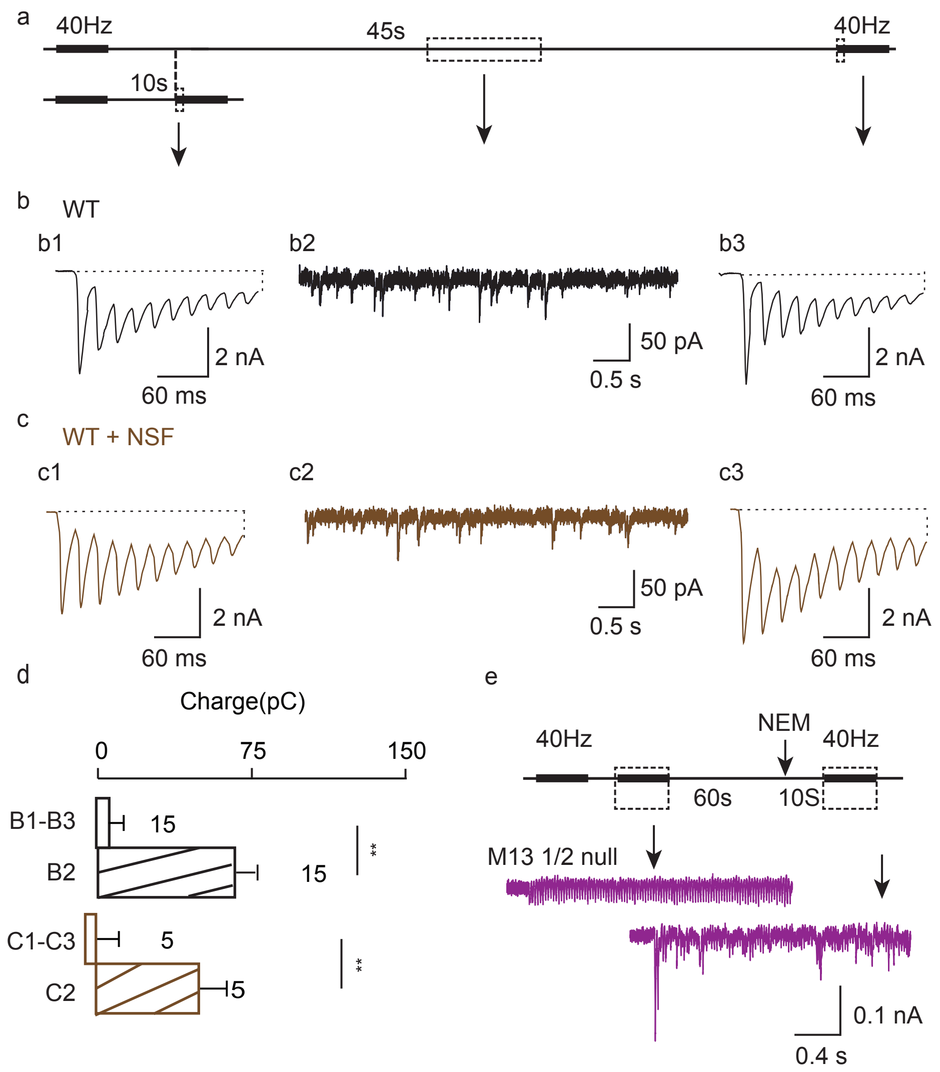

Supplementary Figure3
de-priming is undetected in the WT and NSF over-expressed WT neurons

(a) Paradigm to assess de-priming by dual 40Hz train stimulation (see methods for details).
(b, c) sample traces of dual 100 stimuli at 40Hz with 10s or 45s interval in WT (b) and NSF over-expressed WT (c) synapses.
(d) To quantify de-priming, the total charge of the first 10 responses (b1, b3; c1, c3) of each dual 100 stimuli at 40Hz was quantified. The spontaneous fusion of vesicles in the 10-45s intervals (b2; c2) cannot explain the loss of fusion-competent vesicles after 45s, defined as the difference in total charge between 10s and 45s intervals (b1-b3; c1-c3).
(e) Paradigm and sample traces of munc13-1/2 null with train stimulation and NEM application.
All data in this figure are means ± SEMs; p value determined by Wilcoxon signed rank test. See Table S1 for all values, SEMs and n-numbers plotted in this figure.

Supplementary Figure. 4

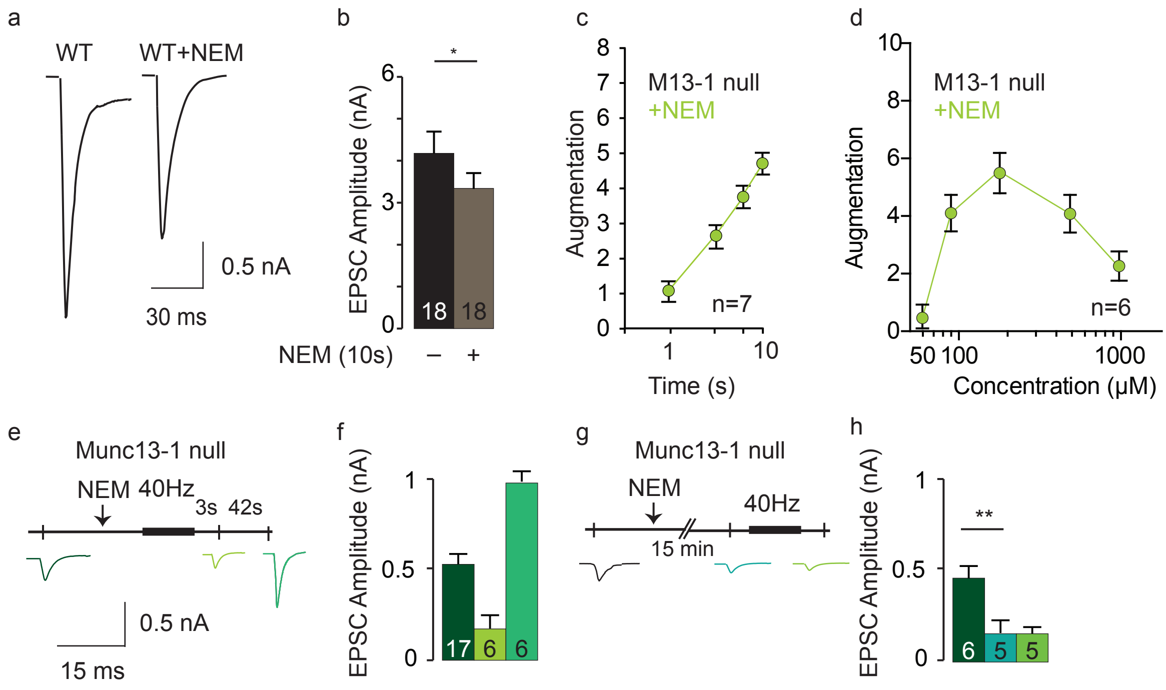

Supplementary Figure 4

NEM effect on evoked EPSC in WT and munc13-1 null autaptic neurons

(a, b) Sample traces (a) and quantification (b) of EPSCs with or without 10 s NEM application.
(c) The effect of NEM on munc13-1 null neurons EPSC builds up during the 1st second after application and reaches highest level within 10s.
(d) NEM potentiates EPSC in a dose-dependent manner.
(e, f) After NEM application, EPSC potentiation recovered in 45 second after 100 stimuli with 40Hz; Paradim and sample trace (e), and quantification (f).
(g, h) After NEM application, EPSC potentiation vanished after 15 min; 100 stimuli at 40Hz cannot re-potentiate EPSC ; Paradigm and sample trace (g), and quantification (h).
p values determined by paired t-test test. See Table S1 for all values, SEMs and n-numbers plotted in this figure.

Supplementary Figure. 5

a

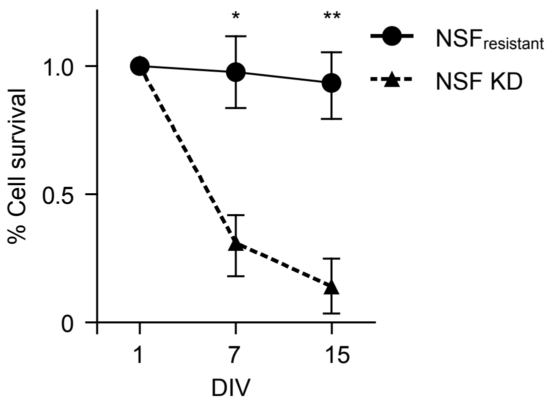

Supplementary Figure 5
NSF knock down by shRNA causes neuronal death

(a) Neuron viability was measured at various time after infection at DIV0. Black circle: neurons with NSF knock down by short hairpins and rescued by shRNA resistant NSF (n=10); Black triangle: neurons with NSF knock down by short hairpins only (n=10). All data in this figure are means ± SEMs;
*p < 0.05, **p < 0.01, ***p < 0.001 as determined by student t-test.

Supplementary Table 1

	M18-1/1SWAP	M18-1/2SWAP	M18-1/3SWAP	M13-1 null	CAPS-1/2 null	M13-1/2 null	WT
number of docked vesicles	6.38±0.26,n=56, fig.1G	4.61±0.26, n=54, fig.1G	2.69±0.23, n=65, fig.1G				
acitve zone size	284.5±15.9 nm, n=56, fig.1H	283.6±17.9 nm, n=54, fig.1H	312.9±16.3 nm, n=65, fig.1H				
total numer of vesicles	79.3±4.7, n=56, fig.1I	72.4±5.2, n=54, fig.1I	72.8±4.8, n=65, fig.1I				
EPSC amplitude	3.7±0.1 nA, n=73, fig.1B	0.42±0.07 nA, n=90, fig.1B	0.1±0.02 nA, n=49, fig.1	0.41±0.08 nA, n=119, fig.3B	0.75±0.19 nA, n=68, fig.3H	0.003±0.003 nA, n=5, fig.3[illegible]	3.8±0.3 nA, n=51, *
	3.1±0.3 nA, n=9, *	0.36±0.13nA, n=48, fig3F		0.51±0.14 nA, n=6, sfig.3F			4.1±0.8 nA, n=18, sfig.3B
EPSC amplitude after 40Hz		1.8±0.3nA, n=48, fig3F		2.1±0.1 nA, n=119, fig.3B	3.4±0.3 nA, n=68, fig.3H		3.4±0.5 nA, n=51, *
EPSC amplitude in presence of 100 μM NEM <10s	2.7±0.3 nA, n=10, *	2.2±0.2 nA, n=23, fig, 3F	0.07±0.03 nA, n=6, *	2.0±0.2 nA, n=41, fig.3B	0.71±0.24 nA, n=17, fig.3H	0.15±0.07 nA, n=19, fig. 3K	3.3±0.6 nA, n=18, sfig.3B
EPSC amplitude in presence of 100 μM NEM >15min				0.6±0.1nA, n=6, sfig.3F			0.2±0.05 nA, n=5, *
mEPSC amplitude	25.4±2.0 pA, n=51, fig.1E	21.7±2.2 pA, n=68, fig.1E	19.1±0.9 pA, n=27, fig.1E	26.1±2.8 pA, n=29, fig.3D	23.8±3.1 pA, n=17, *		22.4±5.1 pA, n=35, *
	21.3±2.5 pA, n=15, *	18.9±3.4 pA, n=20, *					29.1±6.9 pA, n=16, *
mEPSC amplitude in presence of100 μM NEM <10s	24.5±6.2 pA, n=15, *	20±5.5 pA, n=20, *		25.4±3.2 pA, n=29, fig.3D	20.1±2.7 pA, n=17, *		27.6±7.2 pA, n=16, *
mEPSC frequency	17±1.6 Hz, n=51, fig.1D	2.1±0.9 Hz, n=68, fig.1D	0.1±0.04 Hz, n=27, fig.1D	1.6±0.4 Hz, n=29, fig.3D	2.1±0.7 Hz, n=17, *		10.3±4.6 Hz, n=35, *
	16.5±4.1 Hz, n=15, *	2.9±1.2 Hz, n=20, *					8.7±3.9 Hz, n=16, *
mEPSC frequency in presence of 100 μM NEM <10s	12.9±3.3 Hz, n=15, *	7.1±1.5 Hz, n=20, *		4.6±0.6 Hz, n=29, fig.3D	1.2±0.4 Hz, n=17, *		6.1±2.4 Hz, n=16, *
mEPSC charge in 45s-10s		23.9±3.4 pC, n=30, fig.2		20.4±2.8 pC, n=47, fig.2			
first 10 EPSCs charge in second 40 Hz train (45s interval)		51.8±9.0 pC, n=30, fig.2		83.2±8.4 pC, n=47, fig.2			
first 10 EPSCs charge in second 40 Hz train (10s interval)		126.4±11.4 pC, n=30, fig.2		158.5±15.4 pC, n=47, fig.2			
mEPSC charge in 30s-3s		27.9±8.4 pC, n=9, sfig.2		36.1±9.1 pC, n=15, sfig.2			
first 10 EPSCs charge in second 40 Hz train (30s interval)		49.2±11.6 pC, n=9, sfig.2		105.2±12.5 pC, n=15, sfig.2			
first 10 EPSCs charge in second 40 Hz train (3s interval)		155.1±18.8 pC, n=9, sfig.2		199.5±22.7 pC, n=15, sfig.2			
mEPSC charge in 30s-3s				11.7±2.8 pC, n=4, sfig.2			
sucrose charge after 40 Hz train (30s interval)				289.9±42.1 pC, n=4, sfig.2			
sucrose charge after 40 Hz train (3s interval)				371.5±51.4 pC, n=4, sfig.2			

** not shown in figures*
